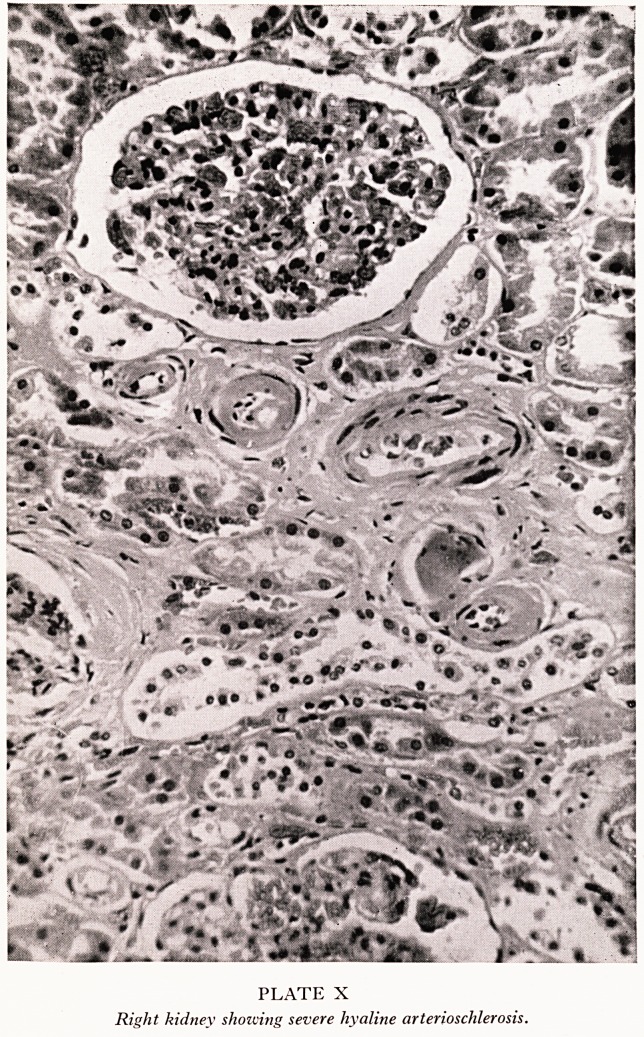# Unilateral Renal Arterial Obstruction

**Published:** 1966-01

**Authors:** O. C. Lloyd


### UNILATERAL RENAL ARTERIAL OBSTRUCTION

*A Clinico-Pathological Conference held in the University of Bristol on 16th March 1965*

*P.M.* 8916

CHAIRMAN: DR. O. C. LLOYD

*Dr. Gough:* Professor Perry, who is out of the country at present, has asked me to come and tell you about this patient of his, a man who was 48 years old when he died, and who worked as a keyboard operator. He was first referred to Professor Perry in January 1961 by his general practitioner. The G.P.'s letter said "He has had the symptoms of cardiac effort pain for two months". The doctor had examined him carefully and found his blood pressure was 240/135. In the same letter the doctor said that there was a very strong family history of cardio-vascular disease, and probably of hypertension also. The patient's father had died at the age of 60 from coronary thrombosis and, of the patient's three brothers, he knew one had hypertension in his early 50's and another had "heart trouble". So his doctor inferred that in a patient over the age of 40 with hypertension and with a strong family history, the hypertension quite possibly fell into the "essential" category.

When Professor Perry saw the patient he agreed completely with the doctor's findings and conclusions. The blood pressure was 240/130 and a significant point was that the fundi showed no hypertensive changes. The femoral pulses were present and were not delayed and therefore coarctation of the aorta was excluded. Professor Perry looked back through the BRI notes and found that the patient had been previously under the care of the surgeons. The patient's first hospital admission had been 18 months before, in September 1959, when he was under the care of Mr. Pocock, with prolapsed and strangulated piles. He was treated symptomatically with good effect and looking back through the surgical notes a surgical dresser, who subsequently became a distinguished houseman and Resident Clinical Pathologist in the BRI, found on routine examination that the blood pressure was 130/80. This point is of great clinical significance, as you will gather as we go along. The patient was discharged and his name placed on the waiting list to come back for haemorrhoidectomy, but it was 8 months later, in June 1960, before this could be arranged. On this occasion his blood pressure was noted to be 240/140, but this was not considered at the time to be of significance, and the operation went ahead as planned, without untoward incident.

I mentioned that it was vitally important to know that the blood pressure was 130/80 only a few months before it was found to be 240/140 because, if the initial reading was correct, it would indicate that some recent factor has been operating, i.e. that there was a "secondary cause" for the sudden rise in blood pressure. The importance of such causes, of course, is that they are often amenable to corrective surgical treatment, e.g. renal artery occlusion or an endocrinal cause such as phaeochromocytoma. Some outpatient investigations were arranged, which included an electrocardiogram, chest X-ray and intravenous pyelogram. Both the ECG and the chest X-ray showed gross evidence of left ventricular hypertrophy and these findings cast a little doubt on the accuracy of the initial normal blood pressure recording by the surgical dresser only a few months before.

If we are to believe this reading, we have to assume that after the normal reading was recorded, the secondary cause of the hypertension began to operate immediately, and in a very few months produced quite gross left ventricular hypertrophy. This possible, but I believe an alternative explanation is that the surgical dresser incorrec recorded the pressure by failing to observe a "silent auscultatory period" which som times occurs in patients with hypertension. After all, he had no reason to susp hypertension in an apparently healthy man with piles and he may have failed to o serve the "golden rule" of first taking the systolic blood pressure by palpation at t wrist. However, all this is conjecture and I must continue with the story. The I.V. done as an outpatient showed no abnormality and there was therefore nothing suggest that the patient had unilateral renal disease or polycystic kidneys as t underlying cause of his hypertension. Meanwhile the patient's name was on the me cal waiting list and it was two months before he could be admitted. On admissio further ECG was taken which showed a marked change from the one taken previou in the Outpatients Department, in that there was now definite evidence of a my cardial infarction. The patient was quite adamant that his symptoms had not chang in any way and that his chest pain only occurred on effort and was promptly relie by rest. His myocardial infarct was therefore a "silent one"—an event that occ in less that 5 per cent of all myocardial infarcts. Other investigations were done an in-patient, most of which were directed towards excluding renal and endocri causes for his hypertension. The urine contained only a trace of albumin, there we no cells in the centrifuged deposit and culture yielded no growth of organisms. T blood urea was 39 mgm/100 ml. Urine concentration test showed only very mi impairment since he was able to concentrate to a maximum of 1020. Urinary e cretion of catechol amines was normal. An aortogram was then arranged and perha at this point Dr. Ross could show us all the radiographs—the chest, the I.V.P. a the aortogram?

*Dr. Lloyd:* One question before Dr. Ross does. You say that his blood pressu in September 1959 was 130/80, in June 1960 the left ventricle was first seen to hypertrophied and he already had clinical hypertension. Is it really surprising th he should have left ventricular hypertrophy in June 1960, if he has already got hype tension?

*Dr. Gough:* Even if we assume that the secondary cause for the hypertension beg to operate immediately after the normal blood pressure recording, this only lea something less that eight months for gross left ventricular hypertrophy to take pla I find this a little surprising.

*Dr. Lloyd:* You do? I had an idea that significant hypertrophy of the left ventri could take place in something like six weeks. Besides which, surely the more rapi the hypertension comes on, the more likely it is that the ventricle will be strained?

*Dr. Gough:* To take your second point first: the electrocardiographic term "l ventricular strain" does not mean the ventricle has been "strained"—such a trac really only indicates a more extreme form of hypertrophy of the left ventricle. Second in response to sudden increases in systematic arterial pressure, I would imagine the l ventricle would first become dilated and then hypertrophy; and that the process initiation of the secondary cause, the elevation of pressure, dilatation and then hype trophy of the left ventricle would take some considerable time.

*Dr. Lloyd:* It doesn't stay dilated because, if it is doing its work properly, it has g to empty itself, and that means it has got to do some more work. Therefore t hypertrophy begins right away.

*Dr. Gough:* Well, perhaps I have judged the surgical dresser too harshly in sayi his B.P. recording might be erroneous, but the doubt must remain until we have t post mortem evidence.

*Dr. Lloyd:* All right. I was not quite sure of the dates, that is why I asked. Yo point is that in the months between September 1959 and June 1960 he had develop

PLATE VII

*Aortogram. Stenosis (arrowed) and post stenotic dilatation of the left renal artery.*

PLATE VIII

*Transverse sections of both renal arteries 0·5 cm, 1 cm and 2 cm beyond their origins (left to right). Artefactual folding of the top left section exaggerates the apparent severity of the stenosis.*

PLATE IX
*Left kidney showing severe hyaline arterioschlerosis.*

PLATE X

*Right kidney showing severe hyaline arteriosclerosis.*

a pronounced degree of left ventricular hypertrophy with hypertension and this makes you wonder whether the blood pressure readings have been correct all along.

*Dr. Gough:* If it is right it is of great importance. Something is happening, something of an acute nature.

*Dr. Lloyd:* Yes, I see. Thank you. Would Dr. Ross now like to show us the arteriograms?

*Dr. Ross:* The X-ray examinations were made between January and April 1961. The chest radiograph showed that the heart was increased in size due to enlargement of the left ventricle. The aorta was unfolded but not dilated. These are the appearances one would expect in a case of systemic hypertension.

The next examination performed was intravenous pyelography. This was done with a view to detecting any indirect evidence of the presence of renal artery stenosis or the presence of any other renal lesion, such as chronic pyelonephritis which could be a causative factor in the production of the hypertension. X-ray contrast media, such as "Hypaque", which is frequently injected in order to perform intravenous pyelography, are excreted unchanged by the glomeruli of the kidney. They are concentrated in the renal tubules by the normal process of water absorption from the glomerular filtrate and excreted with the urine into the calyces. In a kidney whose supplying artery is stenosed, water absorption from the tubules is increased above the normal level. This leads to hyperconcentration of the contrast medium and reduction in the volume of urine produced by the kidney. Therefore, on the X-ray examination one may see some initial delay in excretion of the contrast medium by the affected kidney but as the examination proceeds the density of the contrast medium in the calyces and renal pelvis will be greater on the affected side than on the normal side. In addition, the diminished volume of urine produced by the affected kidney is shown by failure to distend the calyces of the affected kidney so well as on the normal side and the kidney on the side of the renal artery stenosis may be shorter in length (by more than 1·5 cms) than the good kidney. In this patient, the only abnormality shown on the intravenous pyelography was some increased concentration of the contrast medium by the left kidney. This raised the suspicion of renal artery stenosis on the left side and therefore aortography was performed by percutaneous catheterization of the right femoral artery by the Seldinger technique. This revealed atheroma of the right common iliac artery, an aberrant renal artery to the lower pole of the right kidney and a short segment narrowing of the left renal artery just distal to its origin from the aorta associated with post-stenotic dilatation of the artery distal to the narrowed segment (Plate VII). The degree of narrowing amounted to about 60 per cent of the internal diameter of the artery. The appearance of this stenosing indicated that it was due to atheroma. The left kidney was 7 mm longer than the right, as measured during the nephrographic phase of the aortogram.

*Dr. Lloyd:* You say that the diminution of the calibre of the artery at the constricted point was by about 60 per cent. Can you tell us how much reduction in blood flow that means?

*Dr. Ross:* No, I cannot.

*Dr. Lloyd:* Can that be worked out, or is that asking too much?

*Dr. Ross:* The answer is that it can be. Also people now put little catheters through the renal artery stenosis after arteriography and measure the pressure gradient across the stenosis, thereby trying to assess its significance.

*Dr. Lloyd:* Dr. Gough, would you like to go on with the story?

*Dr. Gough:* Professor Perry was most impressed with the aortography films Dr. Ross has shown us and he asked Professor Milnes Walker and Mr. Horton to see the patient with a view to carrying out some form of renal artery reconstruction. If the renal artery stenosis was a real one and the hypertension resulted from ischaemia of the kidney on that side, then that kidney should be capable of normal function since th stenosis would have protected it from the deleterious effects of the raised arteri pressure. Preservation of such a kidney is therefore desirable and the operative repai would be directed towards excising a stenosis or removing an atheromatous plaqu by endarterectomy. However, at a joint medical and surgical discussion it was decide that the operative risk was too great in a man with quite severe hypertension and ischae mic heart disease who had suffered a recent myocardial infarction. It was decide that he should be treated with hypotensive drugs and a low-calorie reducing die Initially he was given reserpine and chlorothiazide and later guanethidine but at n time during the next two or three years that he was followed in Outpatients, did w gain satisfactory control of the blood pressure. We endeavour to maintain the diastoli pressure of hypertensive patients between 90 and 110 mms Hg, but at several recording in Outpatients, this patient's diastolic pressure was always in the range 110–12 mms Hg.

The next significant event in the history took place on 30th November 196 when he was readmitted to the Royal Infirmary as an emergency with a clinical diag nosis of acute myocardial infarction. By the time he arrived in the Casualty Depart ment he was in acute left ventricular failure and he died within 12 hours of admissio

To summarize then, we have a 48 year old man who was found to have a blood pres sure of 240/140 a few months after a normal level had been recorded. Aortograph suggested the presence of renal artery stenosis, and we might postulate that bloo flow past the stenosis was reduced so rendering the kidney ischaemic. Renal ischaem is known to produce hypertension, via the hypertensinogen–angiotensin mechanis which causes a rise in systemic arterial pressure. We know that in addition to hyper tension he also had ischaemic heart disease of such severity that he had two myocardi infarcts—the second one causing his death. However, I have cast some doubt on th sequence of events, and have suggested an alternative explanation, i.e. that in view o his age and strong family history of hypertension, he had had essential hypertensio for some years before his death and that the normal B.P. recording by the surgica dresser was an erroneous recording. We know, of course, that hypertension of lon standing contributes to the advancement of coronary artery disease, and it was th latter which finally caused his death *via* a myocardial infarct.

*Dr. Lloyd:* Dr. Martin, would you like to give us the post mortem findings no and then we can discuss the case as a whole?

*Dr. Martin:* The subject was a heavily built man weighing 97 kg. The hea (weight 670 g) showed gross hypertrophy of the left ventricle. The posterior wall o the left ventricule was fibrosed and thinned—an old myocardial infarct. A sectio taken from the anterior wall of the left ventricle shows a recent myocardial infarct wit an area of necrotic muscle surrounded by granulation tissue.

The renal arteries appeared normal externally, but on section (Plate VIII) the le renal artery can be seen to be narrowed by an atheromatous plaque just beyond it origin. Cross-sections of the artery 1 cm and 2 cm beyond the origin show a moderat poststenotic dilatation. The right renal artery appears normal.

In spite of the unilateral renal artery stenosis the kidneys were of approximatel equal size (left 180 g, 12 cm long; right 175 g, 11·3 cm long). The surfaces and cu surfaces appeared normal. Microscopy of sections from both kidneys shows a simila mild degree of ischaemic change and a similar severe degree of hyaline arteriolo sclerosis (Plates IX and X).

Whether the renal artery stenosis caused his hypertension or was caused by hi hypertension or was unrelated to his hypertension is difficult to say.

*Dr. Lloyd:* A very nice problem. The really important point here is the amount o reduction in blood flow that you get with 60 per cent narrowing. Now I do not kno how much it is, and Dr. Ross says he does not either, but I rather suspect that it may not have been enough to cause a significant degree of ischaemia in that kidney. Dr. Martin pointed out that there does not appear to be any reduction in size of the left kidney, as in ischaemia, that there is not any crowding together of the glomeruli, as a result of loss of parenchyma. But even if there were significant stenosis, the evidence is that it was preceded by the hypertension since the degree of post hypertensive changes one can see in the arterioles can be matched equally on both sides. If the renal artery stenosis had been present before the hypertension, then the left kidney would have been protected by the stenosis from the effects of hypertension to an extent which we might reasonably expect would have produced less in the way of arteriosclerosis on the left side than on the right. So this man's family doctor may have been perfectly right, since there was a familial tendency to get essential hypertension.

*Dr. Knapp:* If the renal artery stenosis was significant and the renin–angiotensin mechanism was activated we might have expected him to have secondary aldosteronism. May I therefore ask if the electrolytes were estimated and ask the pathologists if there was any morbid anatomical evidence of adrenal overactivity?

*Dr. Martin:* The adrenals appeared normal in size; they were not weighed. I noted that they had a thick lipid-laden cortex.

*Dr. Lloyd:* You normally get a thick lipid-laden cortex in hypertension.

*Dr. Gough:* In answer to the first part of Dr. Knapp's question—this man was investigated in 1960 when the relationship of angiotensin to aldosterone secretion had not been substantiated. At this time, therefore, we were not requesting the estimation of serum electrolytes in all cases of hypertension, unless we suspected primary aldosteronism.

*Question:* Why do you get dilatation of the renal artery after the stenosis?

*Dr. Ross:* It is always stated that if you get narrowing in an artery you get blood going through it in a jet through the centre of the stenosis and then you get eddying taking place and this transmits a force laterally and produces post-stenotic dilatation. Whether this is right or not I don't know, but that is the explanation that is given.

*Dr. Gough:* There are two further points I should like to raise. Firstly, I should have mentioned in the clinical findings, that we listened for a renal artery murmur but were unable to hear one. This is a sign we routinely seek in all hypertensive patients. The best site to listen for the murmur is 2 in. lateral to the umbilicus—remembering, of course, to listen on both sides.

Secondly, we must remember that radiological demonstration of renal artery narrowing does not always mean that this is the cause of the hypertension. Indeed, such a radiological sign can be demonstrated in many patients with normal blood pressure. Even when we are dealing with a patient with hypertension, the radiological demonstration of renal artery narrowing is not an absolute indication for surgical intervention, since it has been shown that only about one-third of such patients have an ischaemic kidney on the side of the apparent obstruction. It is possible to detect renal ischaemia by divided renal function tests, since an ischaemic kidney handles water and electrolytes in a highly characteristic way and therefore under ideal conditions such a pattern should always be demonstrated before surgical intervention is attempted.

*Dr. Lloyd:* Would you tell us which patients you must investigate to find out whether there is evidence of unilateral renal disease?

*Dr. Gough:* It is well established that essential hypertension is seldom encountered under the age of 35–40, so therefore we have the highest hopes of demonstrating a secondary cause of hypertension in patients below 40. Every patient under 40 with significant hypertension is most carefully investigated, using all the diagnostic tools at our disposal. I must admit, however, that we by no means always demonstrate a cause of hypertension in our young patients, and at this point I should say that renal artery stenosis as a cause of hypertension is rare in Bristol. Among the many patients that have been investigated by aortography I only know of one, a young soldier of 20, who has had a successful surgical correction of renal artery stenosis with permanent reduction of blood pressure. The more common secondary causes of hypertension in the under 40s are chronic nephritis and, in females particularly, chronic pyelo-nephritis.

*Student:* When is surgical correction of renal artery stenosis attempted?

*Dr. Gough:* Under ideal circumstances the following criteria should be satisfied: a patient with significant hypertension, who has been shown to have radiological evidence of renal artery obstruction at aortography with, usually, a smaller kidney on the affected side; the functional pattern of renal ischaemia should also be demonstrated on the affected side, i.e. a kidney which excretes less water and less sodium chloride than the unaffected kidney—in other words, in the ischaemic kidney there is increased tubular reabsorption of sodium chloride and water. If all these features can be demonstrated then operation has a good chance of success.

*Dr. Enoch:* What about the value of renal biopsy?

*Dr. Gough:* Yes, this is of value. In the presence of hypertension due to renal artery stenosis one would expect the blood vessels in the kidney on the affected side to show a normal histological pattern, since they are protected from high arterial pressure by the stenosis, while on the contralateral side vascular changes may be present, the extent of which will depend upon the duration and severity of the hypertension. If both kidneys show hypertensive vascular changes then operation clearly has no prospect of success.

*Dr. Lloyd:* That concludes a very interesting case.

## Figures and Tables

**PLATE VII f1:**
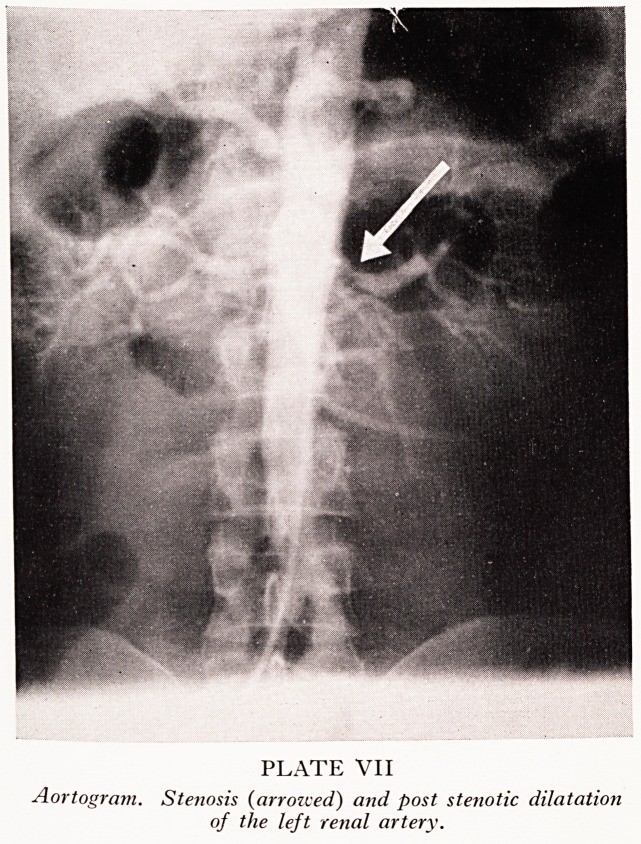


**PLATE VIII f2:**
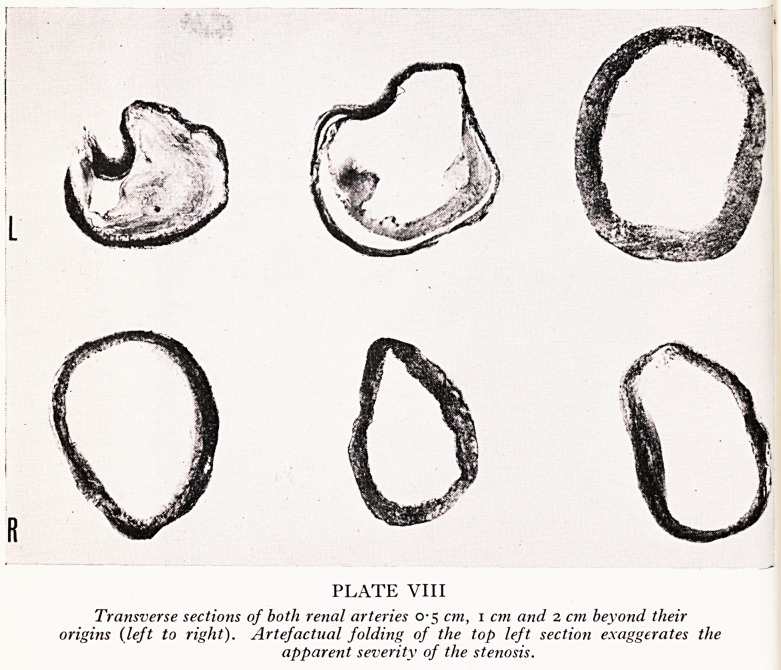


**PLATE IX f3:**
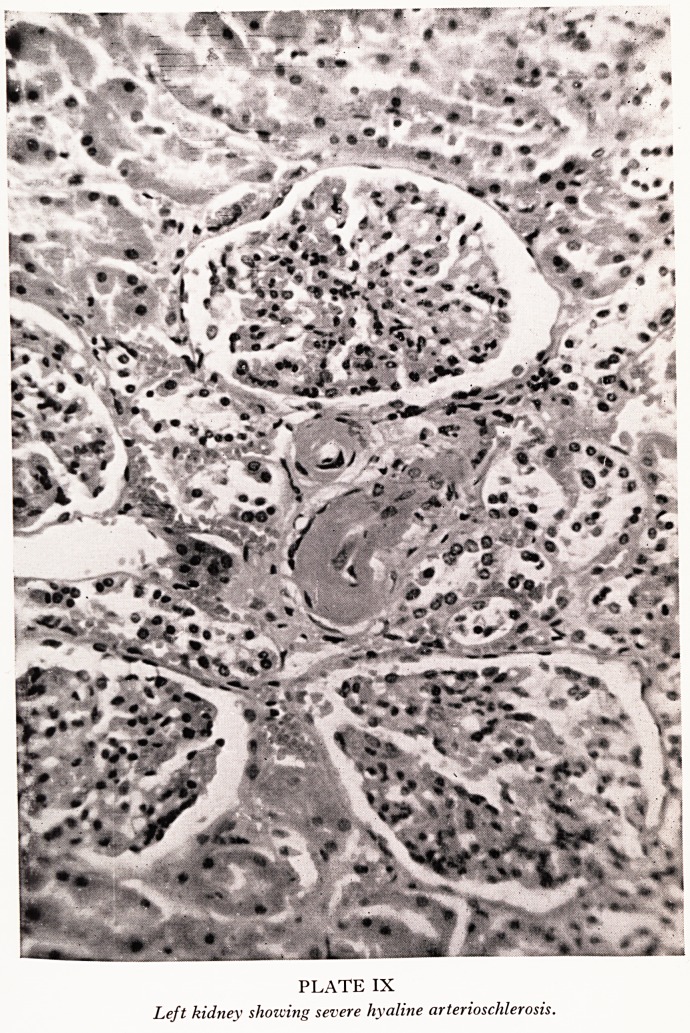


**PLATE X f4:**